# Cell surface dynamics and cellular distribution of endogenous FcRn

**DOI:** 10.1371/journal.pone.0182695

**Published:** 2017-08-17

**Authors:** Lena D’Hooghe, Andrew D. Chalmers, Sam Heywood, Paul Whitley

**Affiliations:** 1 UCB Pharma Ltd, Slough, United Kingdom; 2 Department of Biology and Biochemistry, University of Bath, Bath, United Kingdom; Institut Curie, FRANCE

## Abstract

A major role for FcRn is the salvage of pinocytosed IgG and albumin from a degradative fate in lysosomes. FcRn achieves this by binding IgG in a pH-dependent manner in acidic endosomes and recycling it to the plasma membrane to be released at neutral pH. This is important in maintaining high serum IgG and albumin levels and has the potential to be exploited to modulate the pharmacokinetics of antibody-based therapeutics. Although FcRn is responsible for the recycling of IgG, the dynamic behaviour of endogenous FcRn is not well understood. Our data shows that the majority of endogenous receptor is distributed throughout the endosomal system and is present only at a low percentage on the plasma membrane at steady state. A significant fraction of FcRn at the cell surface appears to be endocytosis resistant while the remainder can undergo rapid endocytosis. To maintain surface levels of the receptor, endocytosed FcRn is replaced with FcRn from the internal pool. This unexpected complexity in FcRn cell surface dynamics has led us to propose a model for FcRn trafficking that should be taken into account when targeting FcRn at the cell surface for therapeutic purposes.

## Introduction

The MHC class-I related neonatal Fc receptor (FcRn) mediates transfer of maternal IgG from parent to offspring, providing passive humoral immunity in early life [1]. FcRn expression is not however restricted to prenatal and young mammals. It is expressed widely in endothelial and epithelial cells and in tissues including liver, kidney and muscle of human adults [2]. In adults, FcRn has a number of proposed functions such as antibody-mediated antigen presentation in dendritic cells [3] and facilitating transcytosis of IgG across epithelial barriers, both from basolateral to apical surfaces and *vice versa* [4]. However, one of the main functions of FcRn in adults is in maintaining serum IgG and albumin levels. Mice with FcRn ‘knocked out’ have markedly reduced serum IgG levels and IgG half-life compared to wild type controls [5]. In humans, siblings with familial hypercatabolic hypoproteinemia [6], a condition characterised by reduced serum IgG and albumin levels with shorter half-life, were discovered to have an FcRn deficiency due to mutation in the β2-microglobulin gene [7], a subunit of FcRn.

FcRn maintains high serum IgG levels by rescuing intracellular IgG, taken up by fluid-phase endocytosis, from degradation in lysosomes [8]. It facilitates this rescue due to its pH-dependent association with IgG [9, 10], that allows binding of IgG in the acidic environment of endosomes and release at the near neutral pH at the cell surface [11]. This salvage of IgG requires that FcRn with bound IgG is transported from an intracellular compartment to the plasma membrane. A number of elegant studies utilising live imaging of endothelial cells transfected with FcRn tagged with a fluorescent protein such as GFP have characterised the site of IgG salvage to be sorting endosomes [12–14]. It seems that IgG bound to FcRn is sorted into tubules originating from sorting endosomes leading to its return to the plasma membrane, while IgG unable to bind FcRn is not sorted into tubules and eventually gets degraded in lysosomes [13]. FcRn protects albumin from catabolism in a similar pH dependent manner to IgG [15], however the albumin and IgG binding sites on FcRn are distinct [16].

The IgG/albumin salvage function of FcRn has been exploited to extend the serum half-life of biologics by fusing Fc or albumin to the active agent. Conversely when a reduction of half-life of endogenous IgG is desirable, such as in the removal of pathogenic autoreactive antibodies, blockade of FcRn (by IVIG) can be performed [17, 18], which essentially blocks the IgG salvage function of FcRn. This blockade approach however is non-specific leading to global depletion IgG and not just the desired species. The aforementioned strategies primarily depend on the binding of FcRn to IgG at acidic pH for their mechanisms of action although AbDegs, engineered to bind FcRn with high affinity at both neutral and acidic pH may have increased efficacy to IVIG [19].

Binding to FcRn at neutral pH also has additional potential therapeutic value. For example ‘sweeping’ antibodies are engineered to bind to FcRn via their Fc domain at neutral as well as acidic pH. Furthermore, their antigen binding site is made pH-dependent so that antigen is bound at neutral pH but released at acidic pH [20]. Thus, sweeping antibodies are proposed to function in the following way. They bind to FcRn and antigen at neutral pH at the cell surface. They get internalised by endocytosis (FcRn- receptor mediated) and release antigen in the acidic environment of endosomes. They get recycled back to the cell surface without antigen but still in complex with FcRn. Once returned to the cell surface they can bind more antigen. The sweeping antibody approach might be useful in depleting soluble antigens such as pro-inflammatory mediators of autoimmune reactions. They have enhanced efficacy in antigen removal over conventional antibodies and pH-dependent antibodies, having the potential to reduce frequency and concentration of dosage [21].

While data from many studies is consistent with pinocytosed IgG being recycled back to the plasma membrane by FcRn, it has not been demonstrated that the FcRn itself is continuously endocytosed from and recycled back to the plasma membrane as is the case for transferrin receptor (TfR) [22]. In order to assess the potential of antibodies, particularly sweeping antibodies [20] that gain enhanced function through trafficking of FcRn, it is necessary to begin to understand something about the kinetics of FcRn internalisation and return to the cell surface.

In this study cell surface biotinylation assays and fluorescence microscopy have been used to investigate the distribution of endogenous FcRn and quantify the proportion that is at the surface of HepG2 cells. The dynamics of removal of FcRn from, and return to, the cell surface have also been investigated, revealing some unexpected behaviour. We present a model for endogenous FcRn trafficking that should be taken into consideration when targeting surface FcRn for therapeutic purposes.

## Materials and methods

### HepG2 cell culture

HepG2 cells (European Collection of Authenticated Cell Cutures) were purchased from Sigma Aldrich (85011430-1VL). HepG2 cells (passage 5–18) were grown in Dulbecco’s Modified Eagle’s Medium (DMEM) supplemented with 10% Foetal Bovine Serum and 2mM L-Glutamine (Gibco, Thermo Fisher Scientific) at 37°C/5% CO_2_. Cells were split 1 in 10 once they reached 70–80% confluency.

### Surface biotinylation of HepG2 cells

2x10^6^ HepG2 cells per well were plated in 10cm^2^ tissue culture plates (BD Falcon) and incubated at 37°C/5% CO_2_ for 1–2 days. Media was removed and cells were washed twice with 5ml PBS/CM (with Ca^2+^ and Mg^2+^) (Sigma). Cells were incubated on ice on a rocking platform with 5ml EZ link sulfo-NHS-SS-biotin (Pierce, Thermo Fisher Scientific) at 0.5mg/ml in PBS/CM for 30 min. The ‘no biotin’ control was incubated with 5ml PBS/CM for 30 min. Cells were then incubated three times with 50mM NH_4_Cl (in PBS/CM) on ice for 5 min to quench excess NHS-SS-biotin. Cells were then washed three times with 5ml PBS/CM.

### Endocytosis assay

Pre-warmed DMEM (complete) was added to surface biotinylated cells and incubated at 37°C for 0 (strip control), 5, 10, 30 and 60 min to allow endocytosis. Cells were transferred onto ice and immediately washed with 5ml ice-cold PBS/CM to stop endocytosis. For surface stripping, cells were incubated three times with 5ml 100mM MESNA in TBS/C (with Ca^2+^) at 4°C for 15 min each to remove biotin from labelled proteins on cell surface. Cells were washed three times with 5ml 5mg/ml Iodoacetamide in PBS/CM at 4°C, each for 5 min, to quench MESNA and then lysed.

### Recycling assay

Pre-warmed DMEM (complete) was added to cells immediately following biotinylation and incubated at 37°C for 30min. Cells were surface stripped with MESNA at 4°C as above. Following this, cells were incubated for a second time at 37°C for 10, 30 and 60 min to allow trafficking to resume. The return of internal biotinylated proteins to the cell surface was then assessed. Cells were transferred onto ice and washed with 5ml ice-cold PBS/CM to stop endocytosis/recycling. Cells were surface stripped for a second time with MESNA at 4°C as above. One sample was not surface stripped for a second after 60 min acting as a degradation control. Finally, cells were washed three times with 5ml 5mg/ml Iodoacetamide in PBS/CM at 4°C for 5 min to quench MESNA and then lysed. Biotinylated FcRn remaining internal following two rounds of surface stripping was recovered and quantified (see below).

### Repeated biotinylation assay

2x10^6^ HepG2 cells were biotinylated for 30 min as described above. Pre-warmed PBS/CM was added to biotinylated cells and incubated at 37°C for 10 min to allow endocytosis. Cells were transferred onto ice and washed with ice-cold PBS/CM to stop endocytosis. Biotinylation and endocytosis steps were repeated for a total of 4 rounds. After each 10 min incubation, one of two plates of cells was surface stripped with 5ml 100mM MESNA (in TBS/C) at 4°C for 15 min. Surface stripped cells were washed three times with 5mg/ml Iodoacetamide in PBS/CM at 4°C for 5 min to quench MESNA and then lysed.

### Cell lysis and isolation of biotinylated protein

Cells were lysed on ice in 800μl lysis buffer (1.25% Triton X-100, 0.25% SDS, 50mM Tris-HCl, pH 8.0, 150mM NaCl, 5mM EDTA, 5mg/ml Iodoacetamide, protease inhibitor) per dish for a minimum of 15 min. Lysed cells were scraped into a microfuge tube and the lysates were sonicated with five brief pulses on ice. Lysates were centrifuged at 15,000*g* (4°C) for 15 min to pellet large/nuclear debris. Supernatants were collected and an aliquot from each dish was used to determine total protein concentration using a BCA assay (Pierce, Thermo Fisher Scientific) and to run on SDS-PAGE for western blotting. 100μl of high capacity NeutrAvidin beads (Pierce, Thermo Fisher Scientific) were added to an equivalent volume of total cell lysate and incubated at 4°C overnight with rotating. Beads were collected by centrifugation at 2000*g* (4°C) for 3 min and washed three times with 1ml wash buffer (0.5% Triton X-100, 0.1% SDS, 50mM Tris-HCl, pH 8.0, 150mM NaCl, 5mM EDTA). Biotin labelled proteins were eluted from the beads with Sample Reducing Agent (NuPAGE, Thermo Fisher Scientific) containing 100mM Dithiothreitol (DTT) in LDS Sample Buffer (NuPAGE, Thermo Fisher Scientific) by boiling at 100°C for 10 min.

### Anti-FcRn western blot and quantification of FcRn by densitometry

Following SDS-PAGE proteins were transferred onto 0.45μm Nitrocellulose membrane (GE Healthcare) in Tris/glycine buffer (25mM Tris and 190mM glycine) at 150mAmps overnight. Membranes were blocked with 5% blocking agent (GE Healthcare) in PBS/0.1% Tween-20 for 1 hour. After blocking, the membranes were washed three times for 5 min with PBS/0.1% Tween-20. Membranes were incubated with a primary antibody: B8 anti-FcRn mouse monoclonal IgG2a (Santa Cruz Biotechnology, sc-271745) diluted 1:1000 in 1% BSA/PBS/0.1% Tween-20 for 1 hour. Membranes were washed six times for 5 min with PBS/0.1% Tween-20 and then incubated with secondary antibody: goat anti-mouse IgG F(ab’)_2_-HRP (Jackson ImmunoResearch Laboratories, Stratech, 115-036-072) diluted 1:2000 for 1 hour. Membranes were washed six times for 5 min before being treated with ECL Select (GE Healthcare). Immunoblots were visualised using a charge-coupled device (CCD) camera system; Image Quant (GE Healthcare). Quantification of FcRn was carried out using Image Quant TL software (GE Healthcare). A standard curve was generated from known amounts of purified recombinant human FcRn extracellular domain (ECD) (courtesy of UCB). The difference in molecular weight between the ECD and endogenous FcRn was taken into account in the quantification.

#### Determining surface FcRn levels using confocal microscopy

HepG2 cells (1x10^4^ per well) were plated onto Biocoat poly-D-lysine chamber slides (BD Biosciences) and incubated at 37°C/5% CO_2_ overnight. Media was removed and cells were washed three times with ice-cold PBS/CM on ice. Cells were fixed in 4% formaldehyde (Pierce, Thermo Fisher Scientific) diluted in PBS/CM for 15 min on ice, followed by 15 min at room temperature. Cells were washed three times with PBS/CM followed by one wash in 0.05M Glycine in PBS/CM for 5 min to quench. For non-permeabilised cells, blocking was with 5% BSA in PBS/CM for 20 min. For permeabilised cells, blocking was with 5% BSA and 0.1% saponin in PBS/CM for 20 min. Cells were incubated with anti-FcRn mIgG1 at 5μg/ml (UCB) and antibodies against transferrin receptor (TfR), early endosome antigen 1 (EEA1), cation independent mannose-6-phosphate receptor (CI-M6PR), trans-golgi network (TGN46) and lysosomal-associated membrane protein 1 (LAMP1). The cell markers were used at 1:200 (TfR, EEA1, CI-M6PR and LAMP1) or 1:400 (TGN46) diluted in 2% BSA/PBS. Cells were washed three times with PBS/CM. Cells (permeabilised and non-permeabilised) were incubated for 20 min with 5% BSA and 0.1% saponin in PBS/CM. Cells were incubated with anti-FcRn mIgG1 (UCB) at 5μg/ml and antibodies against transferrin receptor (TfR) (Abcam #ab84036), early endosome antigen 1 (EEA1) (BD Biosciences #610457), cation independent mannose-6-phosphate receptor (CI-M6PR) (Abcam #ab2733), trans-golgi network (TGN46) (Abcam #ab50595) and lysosomal-associated membrane protein 1 (LAMP1) (Abcam #ab25630). The cell markers were used at 1:200 (TfR, EEA1, CI-M6PR and LAMP1) or 1:400 (TGN46) diluted in 2% BSA/PBS. Cells were washed three times with PBS/CM. Cells (permeabilised and non-permeabilised) were incubated for 20 min with 5% BSA and 0.1% saponin in PBS/CM. Cells were incubated with Alexa Fluor conjugated secondary antibody (Molecular Probes, Thermo Fisher Scientific) diluted 1:200 (goat-anti-mouse/rabbit Alexa Fluor 568) or 1:400 (goat-anti-human Alexa Fluor 488) in 2% BSA in PBS/CM. Samples were mounted using ProLong Gold antifade reagent with DAPI (Molecular Probes, Thermo Fisher Scientific).

Confocal microscope images were obtained with 63.0x objective lens with oil immersion using either a Leica TCS-SP5 with Leica LAS AF software (Leica Microsystems) or a Zeiss LSM510META LSM Image Examiner software (Carl Zeiss Microscopy). Co-localisation analysis was performed using Image J software with JACoP plugin. Definiens Architect XD software was used to quantify FcRn levels.

### Statistics

All tests were performed in the GraphPad Prism statistical software package using linear regression (GraphPad Software, La Jolla, California, USA)

## Results

### Distribution of FcRn in HepG2 cells

**Fig 1:**
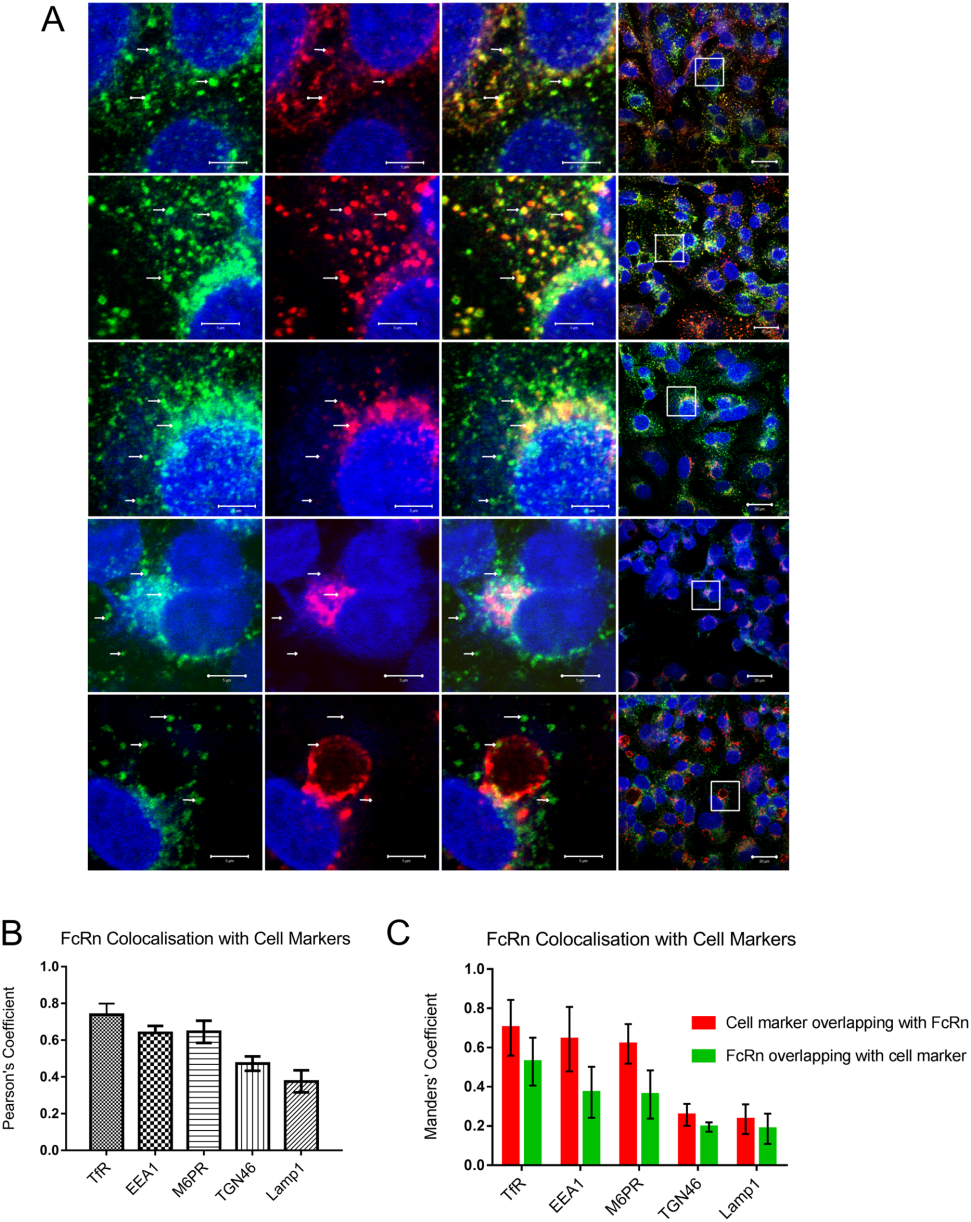
Analysis of co-localisation of FcRn with cellular markers. (A) HepG2 cells were fixed, permeabilised and co-stained with anti-FcRn humanised Fab’, followed by anti-human IgG conjugated Alexa Fluor 488 (first column. Bar 5μm) and antibodies (anti-TFR rabbit IgG, anti-EEA1 mouse IgG, anti-CI-M6PR mouse IgG, anti-TGN46 rabbit IgG and anti-LAMP1 rabbit IgG) against organellar markers followed by anti-rabbit/mouse conjugated Alexa Fluor 568. FcRn fluorescence is shown in green and marker fluorescence is shown in red, yellow fluorescence in the overlay images indicates co-localisation. Images are magnifications of the white boxed area (final column. Bar 20μm). Degree of co-localisation was determined by (B) Pearson correlation coefficient and (C) Manders' overlap coefficients. Co-localisation analysis was performed on a minimum of 3 fields of view each containing approximately 40 cells from 2 independent experiments.

The HepG2 cell line, a human liver hepatocellular carcinoma cell line with an epithelial morphology, was chosen as a model due to the liver being a major site of IgG catabolism [23]. In order to determine the cellular distribution of endogenous FcRn, permeabilised HepG2 cells were immunostained with anti-FcRn antibody and visualised by confocal microscopy (Fig 1). The majority of the FcRn staining was associated with punctate structures throughout the cell, with a greater concentration around the nucleus. Dual labelling with markers for intracellular organelles of the endo-lysosomal system and quantification of co-localisation revealed that FcRn was localised mainly to TfR, early endosome autoantigen-1 (EEA1) and cation-independent mannose-6-phosphate receptor (CI-M6PR) positive compartments. Co-localisation of FcRn to the trans-golgi network (TGN46 positive compartments) and lysosomes (LAMP-1 positive compartments) was relatively low. Therefore, we conclude that endogenous FcRn in HepG2 cells is likely to be mainly distributed in early/late/recycling endosomes but not in the TGN or lysosomes.

**Fig 2:**
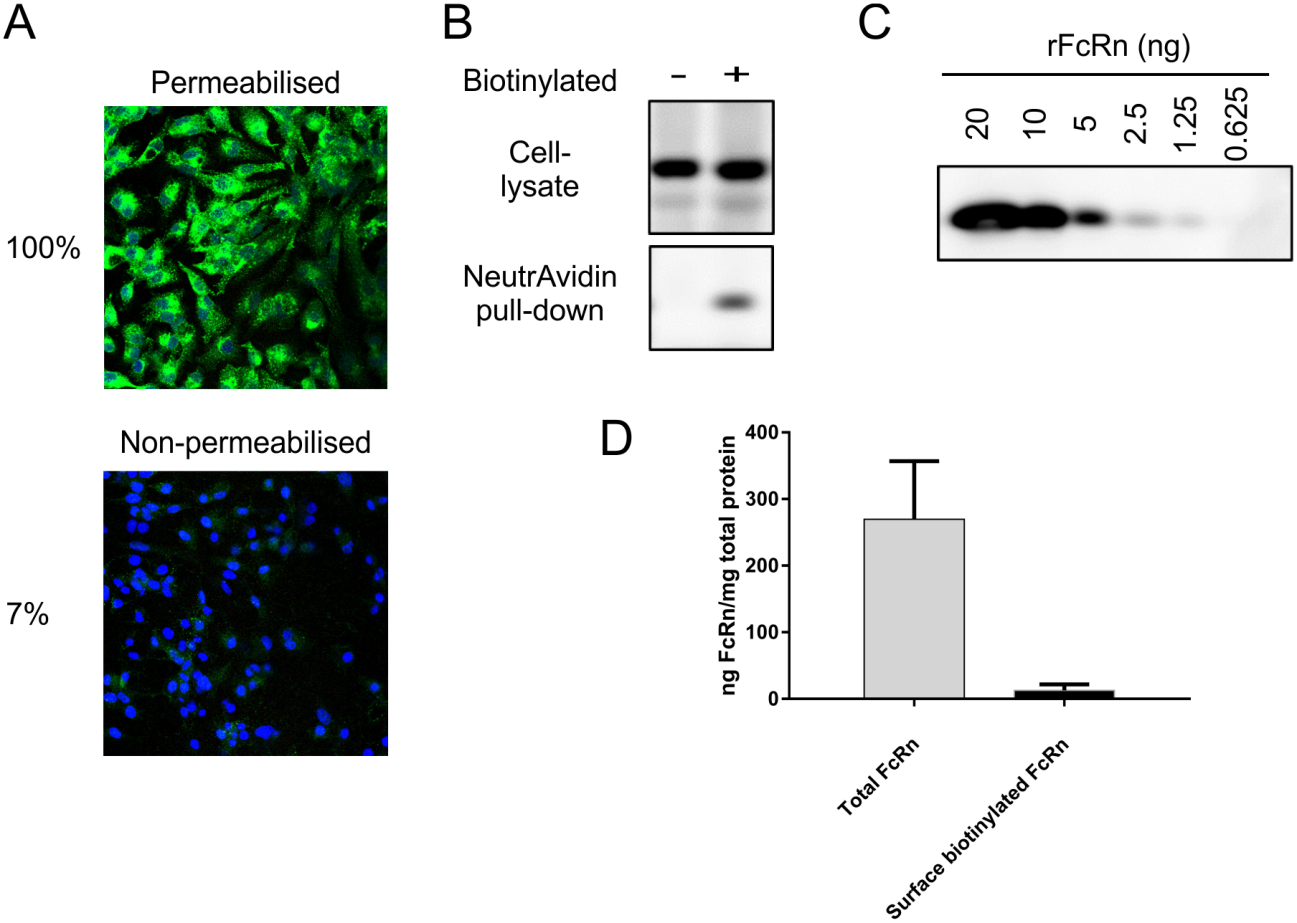
Quantification of surface FcRn. (A) HepG2 cells were fixed and either permeabilised or left untreated. Cells were incubated with anti-FcRn humanised Fab’, followed by goat-anti-human IgG conjugated Alexa Fluor 488 (green). All cells were permeabilised and the nuclei stained with DAPI (blue). Total FcRn levels in non-permeabilised and permeabilised cells determined by measuring intensity of fluorescence over total image (z-stack) divided by the number of cells, as calculated by DAPI stained nuclei, using Definiens software. Fluorescence from permeabilised cells was taken as the 100% value. (B) HepG2 cells were surface labelled with sulfo-NHS-S-S-biotin (+) or left untreated (-). Cells were lysed and incubated with NeutrAvidin beads. The beads were washed and proteins were eluted using DTT and separated by SDS-PAGE. Proteins were transferred onto nitrocellulose membrane and immunoblotted for FcRn. Total FcRn levels in the whole cell lysate of unbiotinylated and biotinylated cells and in the NeutrAvidin pull downs were determined by densitometry. Amounts of FcRn in each sample were extrapolated from a standard curve of (C) known amounts of recombinant FcRn run on the same gel. Note: Representative blots are shown. Gel from which lanes are extracted are shown in (S1 Fig). Histogram showing results of quantification of eight independent experiments. Mean ± standard deviation are shown.

It was not possible to determine the proportion of FcRn at the plasma membrane from immunostaining of permeabilised cells alone. Therefore, permeabilised and non-permeabilised cells were immunostained for FcRn and a comparison made (Fig 2A). Weak, but detectable fluorescence was observed on the surface of non-permeabilised cells (Fig 2A). The total fluorescence associated with permeabilised and non-permeabilised HepG2 cells following FcRn immunostaining was quantified (Fig 2A). Non-permeabilised cells had 7% of the fluorescence of permeabilised cells, representing the percentage of total cellular FcRn present at the plasma membrane.

**Fig 3:**
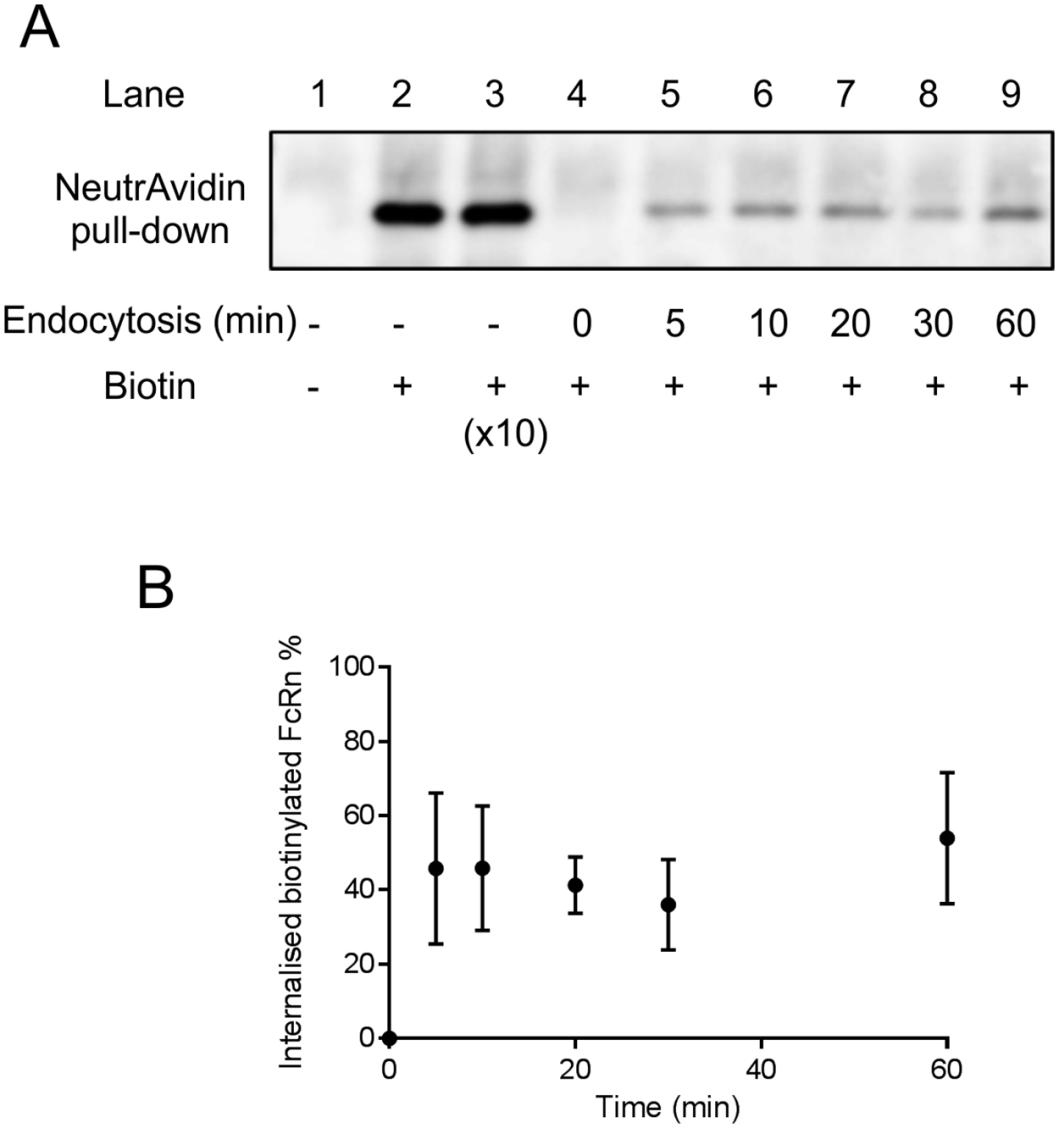
Measuring the rate of endocytosis of surface biotinylated FcRn in HepG2 cells. (A) Cells were surface labelled with sulfo-NHS-S-S-biotin, incubated at 37°C for 0-60min. Surface biotin was stripped from protein at indicated time with a cell impermeable reducing agent. Cells were lysed and incubated with NeutrAvidin beads. The beads were washed and proteins were eluted using DTT and separated by SDS-PAGE. Proteins were transferred onto nitrocellulose membrane and immunoblotted for FcRn. The anti-FcRn immunoblot shows the NeutrAvidin bead eluates. To ensure the amounts of biotin were not a limiting factor in surface labelling, sulfo-NHS-S-S-biotin at 0.5mg/ml and 5mg/ml was used (lane 2 and 3). Lane 4 is the strip control (0 min internalisation) to show the efficiency of stripping biotin from surface exposed FcRn. Lanes 5–9 are samples that have been incubated for 5, 10, 20, 30, 60 min respectively and show internal biotinylated FcRn that is resistant to surface stripping. (B) Quantification of biotinylated FcRn that is resistant to surface stripping at indicated time points. The mean ± standard deviation of four independent experiments are shown. No significant difference is seen between5 and 60 min as analysed by linear regression.

A surface biotinylation assay was also used as an alternative method of quantifying the plasma membrane associated FcRn. The quantity of biotinylated (surface) FcRn recovered by NeutrAvidin pull-down was compared to total cellular FcRn by SDS-PAGE followed by immunoblotting (Fig 2B). Known amounts of recombinant FcRn extracellular domain present on the same gel were used to quantify endogenous FcRn (Fig 2C). Quantification by densitometry of immunoblots revealed that 4% of total cellular FcRn was biotinylated by the membrane impermeant biotin reagent (Fig 2B and 2D). Importantly, the amount of biotinylation reagent used was not limiting as even when its concentration was increased tenfold there was no increase in the amount of FcRn being labelled on the surface of cells (Fig 3A). Thus, quantification using this method estimates approximately 4% of the total cellular FcRn at the plasma membrane.

Two independent methods show the percentage of endogenous plasma membrane associated FcRn to be well below 10% with greater than 90% of FcRn associated with intracellular, mainly endosomal compartments.

### A pool of surface FcRn is rapidly endocytosed

**Fig 4:**
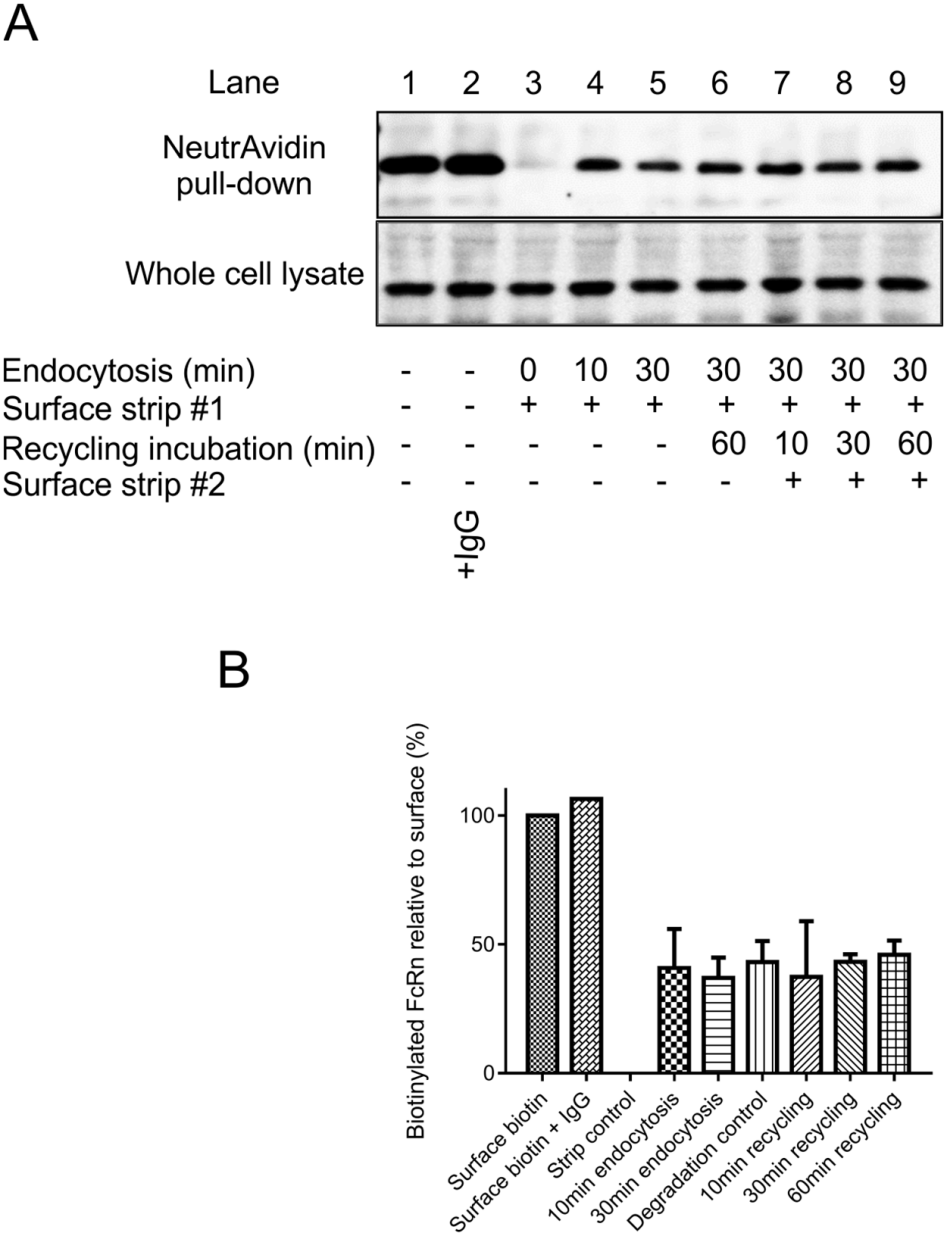
FcRn recycling. (A) HepG2 cells were cultured in the presence (lane 2) or absence of IgG (all other lanes). Cells (lanes 1–9) were surface labelled with sulfo-NHS-S-S-biotin. Cells (lanes 3–9) were incubated at 37°C for indicated (endocytosis) time. Remaining surface biotin was stripped from proteins with a cell impermeable reducing agent. Cells incubated for 30 min at 37°C followed by surface stripping (lanes 6–9) were incubated for a second time at 37°C (recycling incubation) for indicated time and surface stripped for a second time where indicated (lanes 7–9, surface strip #2). A degradation control (lane 6) was incubated for a second time at 37°C for 60 min but was not treated with reducing agent for a second time. Cells were lysed and incubated with NeutrAvidin beads. The beads were washed, proteins were eluted using DTT and then separated by SDS-PAGE. Proteins were transferred onto nitrocellulose membrane and immunoblotted for FcRn. The anti-FcRn immunoblots shows the whole cell lysates (bottom panel) and corresponding NeutrAvidin bead eluates (top panel). (B) A histogram showing the relative amounts of biotinylated FcRn remaining following the indicated treatments. Mean ± standard deviation of two independent experiments are shown. No significant difference between any of the recycling time points tested is seen when linear regression is performed.

To investigate the dynamics of internalisation of the plasma membrane associated pool of FcRn, a surface biotinylation endocytosis assay was utilised [24, 25]. Protection of biotinylated FcRn from ‘stripping’ with a membrane impermeant reducing reagent (which removes the biotin reagent from cell surface proteins) is a measure of internalisation. Surface biotinylated FcRn was rapidly protected from surface stripping, reaching a maximum level of protection (approximately 50% protected in 5 minutes (Fig 3A and 3B). Surprisingly, the level of protection was constant independently of when the stripping was performed after 5 minutes up to 60 minutes. Of note is that total biotinylated FcRn is not detectably reduced when cells are incubated for 1 hour at 37°C (Fig 4A). This indicates that any reduction in amount of biotinylated FcRn recovered in comparison to non-stripped controls is due to biotin removal by the stripping reaction and not due to internalised biotinylated FcRn being degraded in lysosomes.

### Endocytosed FcRn is not rapidly recycled back to the cell surface

The results of the endocytosis assay could be interpreted as 50% of surface biotinylated FcRn being rapidly endocytosed and protected from stripping by 5 minutes with the other 50% not being endocytosed, even after an hour. However, an alternative interpretation is that by 5 minutes, trafficking has reached an equilibrium where the amount of biotinylated FcRn returning to the plasma membrane balances the amount being endocytosed with 50% of the biotinylated FcRn being at the cell surface at any given time point. In order to test which is the most likely scenario an endocytosis/ recycling assay was performed. In this assay, the return of previously internalised biotinylated FcRn to the cell surface is monitored by introducing a second surface strip at subsequent time points following an additional incubation of cells at 37°C. No loss of biotinylated FcRn was observed upon stripping a second time, independently of the time point tested up to one hour (Fig 4A and 4B). Thus there is no detectable amount of endocytosed (biotinylated) FcRn returning to the cell surface.

The trafficking of many receptors can be influenced by ligand binding. The presence of IgG could mobilise an intracellular pool of FcRn to the cell surface. In order to investigate this possibility, HepG2 cells were incubated with human IgG (1 mg/ml) for 4 hours at 37°C to allow uptake by pinocytosis prior to determining surface levels of FcRn (Fig 4A). The levels of surface FcRn were not altered following this incubation suggesting IgG does not lead to large scale movement of FcRn from intracellular stores to the plasma membrane.

### Surface FcRn is replenished from an internal pool

**Fig 5:**
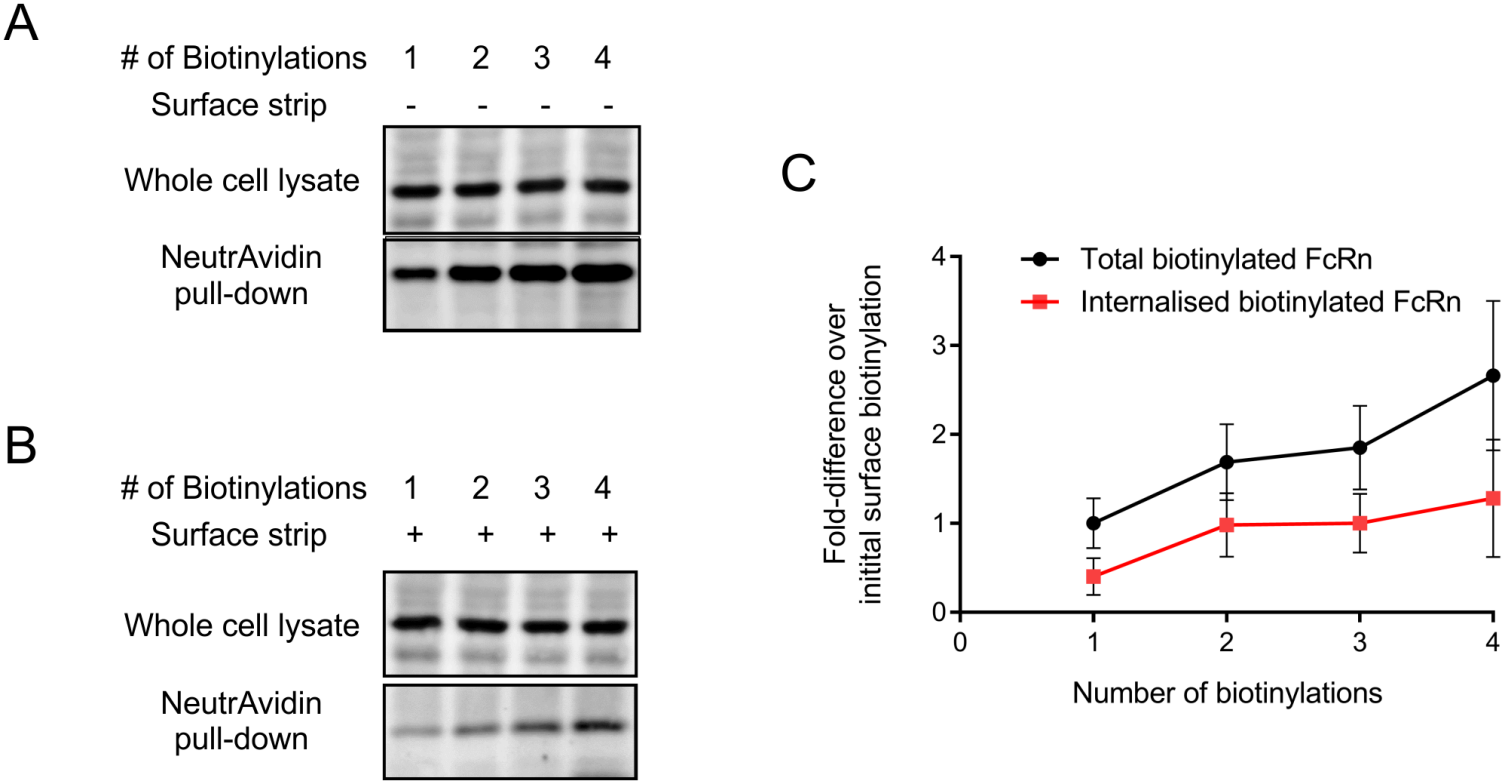
Repeated surface biotinylation. (A) HepG2 cells were surface labelled with sulfo-NHS-S-S-biotin, followed by incubation at 37°C for 10 min (lane 1–4). Cells (lane 2–4) were re-biotinylated and incubated at 37°C for a further 10 min. Cells (lanes 3–4) were re-biotinylated and incubated for a further 10 min at 37°C once and twice respectively. After incubation at 37°C cells were lysed and incubated with NeutrAvidin beads. The beads were washed, proteins were eluted using DTT and then separated by SDS-PAGE. Proteins were transferred onto nitrocellulose membrane and immunoblotted for FcRn. The anti-FcRn immunoblots shows the whole cell lysates (top panel) and corresponding NeutrAvidin bead eluates (bottom panel). (B) Cells were treated as above except rather than cells being lysed immediately following 37°C incubation they were placed at 4°C and treated with cell impermeable reducing reagent. (C) Quantification of biotinylated FcRn following different treatments. The mean ± standard deviation of three independent experiments are shown. Linear regression analysis reveals significant increase in total biotinylated (p = 0.003) and internalised biotinylated (p = 0.0283) FcRn upon increasing rounds of biotinylation.

The results above indicate that approximately 50% of surface FcRn is rapidly endocytosed, but once internalised, is not rapidly recycled back to the plasma membrane. However, as the amount of FcRn at the cell surface should not fluctuate under steady-state conditions, the endocytosed FcRn must presumably be replenished. As greater than 90% of FcRn is in an internal pool it may be assumed that this internal pool or *de novo* synthesised FcRn is responsible for replacing the endocytosed FcRn at the cell surface. In order to test whether this is the case and that the internal pool (or *de novo* synthesised) of FcRn (not biotinylated in first labelling) does indeed have access to the cell surface, an assay was employed in which cells were exposed to multiple rounds of biotinylation (labelling at 4°C), each following a 10 minute incubation period at 37°C to allow for trafficking. The results of this assay reveal that as the number of rounds (up to 4 in total) of labelling with biotin is significantly increased the quantity of biotinylated FcRn is also increased (Fig 5). Furthermore, stripping of cell surface biotin following each additional biotinylation reaction revealed a significant increase in the total pool of biotinylated FcRn protected from stripping. We interpret these results as there being a continuous delivery of FcRn from an internal pool, previously not biotinylated, to the cell surface and subsequent endocytosis of this newly biotinylated FcRn into internal cellular compartments protected from the stripping reagent.

## Discussion

Previous studies have investigated the endosomal trafficking of recombinant tagged FcRn [12–14]. Here, we have investigated the subcellular distribution, cell surface levels and surface dynamics of endogenous FcRn in HepG2 cells.

Endogenous FcRn was found distributed throughout organelles of the endosomal system mainly co-localising with TfR which recycles between the plasma membrane and endosomes [26], EEA1 which is a marker for early endosomes [27] and CI-M6PR which cycles between endosomes and the TGN [28]. It should be noted that quantifying co-localisation is not straightforward [29], although we have taken an unbiased approach by presenting Pearson coefficients and Manders coefficients (with the same threshold values for all images) calculated using the ImageJ Plugin JaCoP [30] from multiple images. Furthermore, the markers used should not be considered as markers of unique compartments [26] with their steady-state distributions almost certainly overlapping in cells. Despite these caveats the cellular distribution of endogenous FcRn in HepG2 cells is consistent with previous reports investigating the distribution of recombinant tagged-FcRn in endothelial cells [13].

The proportion of surface FcRn in HepG2 cells is approximately 4%-7% of the total cellular FcRn. This is very low when compared to TfR in HepG2 cells, which has 33% at the cell surface [31]. However this FcRn distribution is similar to estimates from other cell types such as jejunal epithelial cells of neonatal rat [32] indicating that mechanisms controlling this distribution may be common to different cell types and that HepG2 cells are unremarkable. Cell type specific differences in distribution of FcRn cannot however be ruled out and multiple cell types of different origins that express endogenous FcRn should be tested before generalisations can be made.

Endocytosis of surface FcRn is rapid with around 50% of surface biotinylated FcRn protected from stripping with a membrane impermeant reducing reagent after 5 minutes and does not increase even after 1 hour of endocytosis. This unexpected result supports the presence of an endocytosis resistant pool of FcRn at the plasma membrane of HepG2 cells. If newly endocytosed biotinylated FcRn were recycled directly back to the plasma membrane establishing an equilibrium between endocytosis and exocytosis of biotinylated FcRn this would have been detected in the recycling assay (Fig 4). Using this assay we have readily detected rapid recycling of claudin-1 and transferrin receptor in epithelial cells [24, 33]. Almost identical kinetics of endocytosis of Fab fragments bound to a chimeric FcRn (cytoplasmic domain)/FcγRII (extracellular and transmembrane domain) expressed in MDCK cells have been observed previously, intriguingly with only 50% of surface bound Fab being endocytosed [34]. Together with our data this suggests that there may be some sequence motif in the FcRn cytoplasmic domain responsible for maintaining a balance between endocytosis resistance and competence. The mechanism of retention of the endocytosis resistant surface pool of FcRn remains to be determined but the presence of endocytic resistant pools of receptors at the plasma membrane is not without precedent. Dopamine transporters in the plasma membrane of a human neuroblastoma cell line have been shown to be present endocytosis resistant and endocytosis ‘willing’ populations [35]. Furthermore receptor tyrosine kinases present in actin based plasma membrane protrusions or caveolae are endocytosis resistant [36–38]. It is possible that a pool of FcRn in the plasma membrane of HepG2 cells is immobilised and rendered endocytosis incompetent by cytoskeletal attachment or association with membrane subdomains such as lipid rafts although this will require further investigation. A physiological function of endocytosis resistant FcRn is difficult to speculate on as it doesn’t bind to its known ligands IgG or albumin at neutral pH. However, if endocytosis resistant pools of FcRn are present *in vivo* this is likely to influence the effectiveness of engineered antibodies such as sweeping antibodies that bind to FcRn at neutral pH at the cell surface.

The pool of rapidly endocytosed biotinylated FcRn is replaced by FcRn from an internal pool or *de novo* synthesised that has not been previously biotinylated (Fig 5). With each round of biotinylation more FcRn was labelled with no evidence of plateauing. We propose that given time the entire intracellular pool would eventually have access to the plasma membrane although it was not technically practical to carry out more than 4 rounds of biotinylation. For this reason we cannot rule out the possibility that there is an intracellular pool of FcRn that does not have access to the plasma membrane and is present in a ‘storage compartment’. This ‘stored’ FcRn could be mobilised to the plasma membrane in response to a stimulus such as the presence of high levels of IgG, although we (Fig 4) and others [39, 40] have not detected changes in FcRn subcellular distribution in the presence or absence of IgG. Thus, if there is some signal leading to the mobilisation of intracellular FcRn to the cell surface, or between intracellular compartments, it is yet to be elucidated. FcRn associated with antibodies bound to multivalent antigens do however get sorted to lysosomes demonstrating that trafficking of FcRn can be influenced by extracytoplasmic molecular interactions, possibly due to physical exclusion of large complexes from ‘recycling’ tubules [41].

**Fig 6:**
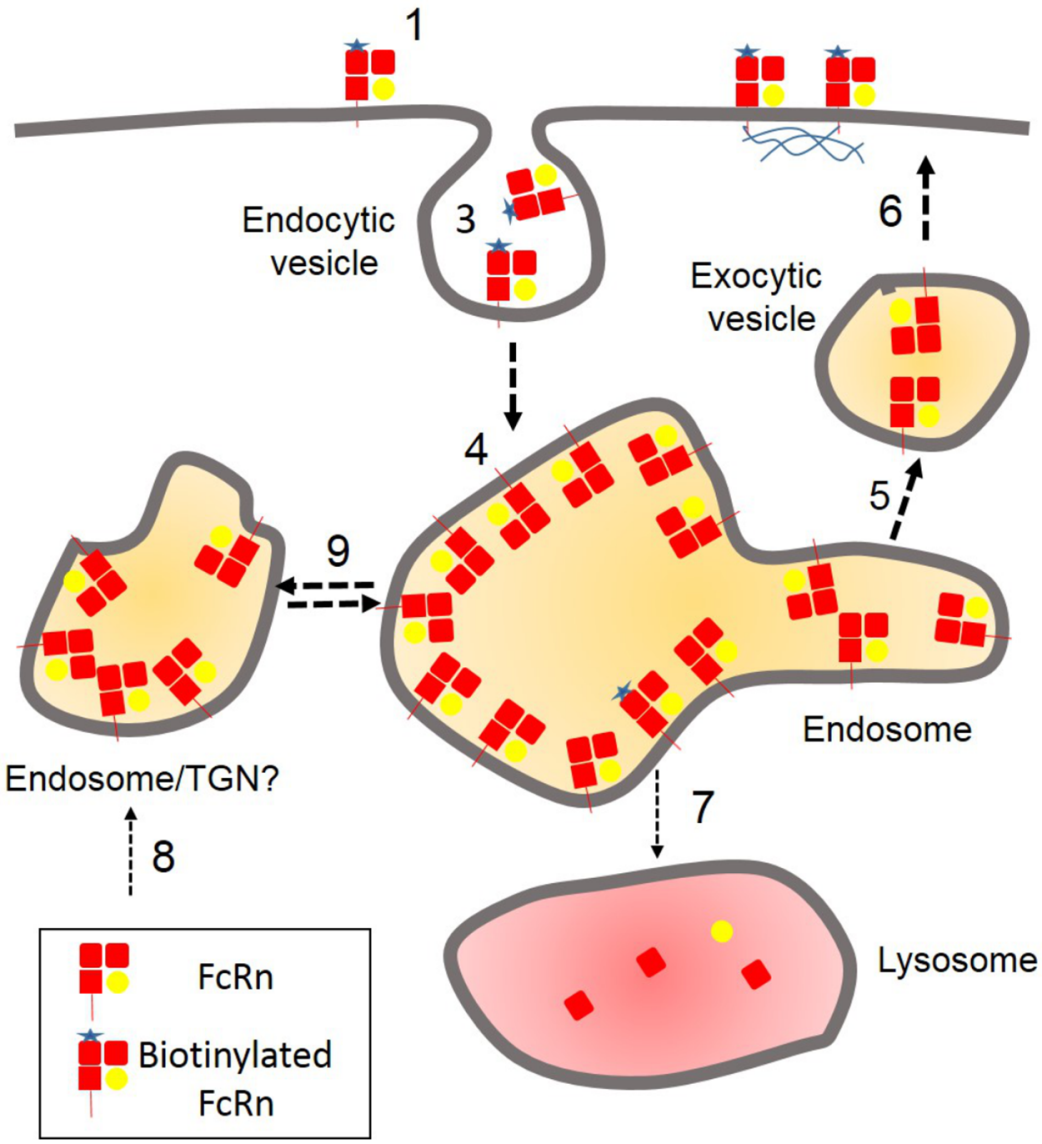
A model for the endocytic trafficking of FcRn. (1) Approximately 4% of cellular FcRn is present on the plasma membrane and accessible to biotinylation reagent. (2) 50% of this surface pool of FcRn is endocytosis resistant possibly due to interaction with cytoskeleton (see Discussion). (3) 50% is rapidly endocytosed and (4) mixes with 96% of cellular FcRn in an internal pool. (5) FcRn from the internal pool (6) replaces endocytosed FcRn at the cell surface to maintain constant levels of FcRn. (7) The trafficking of FcRn to lysosomes for degradation is negligible (not detectable for surface biotinylated FcRn in the time course of our experiments). (80) Any degraded FcRn will be replenished by *de novo* synthesis. (9) There may be exchange between internal pools of FcRn. Note: in this model, mixing of the newly endocytosed biotinylated FcRn (2% of total) with internal pool of non biotinylated FcRn (96% of total FcRn) would mean that less than 2% of FcRn packaged into exocytic vesicles returning to the plasma membrane would be biotinylated. This would not be detectable in our recycling assay.

We propose a model (Fig 6), in which there is continuous exchange between a mobile pool of FcRn on the cell surface (50% is endocytosis resistant) and intracellular FcRn. Consequently each individual FcRn will, on average, reside at least twentyfold longer in an intracellular compartment than at the cell surface due to the intacellular pool being more than twentyfold that of the mobile surface pool. This differs from the classical recycling behaviour of TfR and LDL receptor that rapidly return to the plasma membrane following endocytosis [26]. Although the recycling assay employed does not reveal any return of endocytosed receptor back to the cell surface does not necessarily mean that FcRn does not recycle. With surface derived FcRn being ‘diluted’ by mixing with the large internal pool of FcRn any return of biotinylated FcRn to the cell surface is likely to be below the detection threshold of the methodology that readily detects recycling of TfR. We favour a model in which, on average, each mobile FcRn can cycle multiple times between the plasma membrane and the internal pool, albeit infrequently. This is supported by the observation that surface biotinylated FcRn does not get detectably degraded upon incubation of cells at 37°C. The ‘infrequent’ return of endocytosed FcRn to the cell surface also has implications for sweeping antibodies that bind to FcRn at the cell surface. These will presumably be recycled with the same kinetics as the unbound receptor. The model we present should be further scrutinised experimentally and tested to determine whether it is generally applicable to other cell types.

In summary, our data adds important insight into the dynamic trafficking behaviour of endogenous FcRn from and to the surface of HepG2 cells. Both the presence of an endocytosis resistant pool of surface FcRn and the mixing of endocytosed FcRn with the much greater intracellular pool prior to recycling should be taken into account when designing therapeutic strategies based on binding to FcRn at the cell surface.

## Supporting information

S1 Fig Representative (uncropped) blot from which cropped images in Fig 2B and 2C are taken. Boxed regions correspond to cropped images in Fig 2. Note that the recombinant FcRn is the extracellular domain only and is therefore lower molecular weight than endogenous full length FcRn. This difference is accounted for in calculations.
(PDF)
