# Supplementary material for: Cell surface dynamics and cellular distribution of endogenous FcRn
Source: PLoS One. 2017 Aug 17;12(8):e0182695. doi: 10.1371/journal.pone.0182695 (PMC5560688; doi:10.1371/journal.pone.0182695)
Supplement: S1 Fig — Boxed regions correspond to cropped images in Fig 2. Note that the recombinant FcRn is the extracellular domain only and is therefore lower molecular weight than endogenous full length FcRn. This difference is accounted for in calculations. (PDF) [file pone.0182695.s001.pdf]

Supplementary figure 1

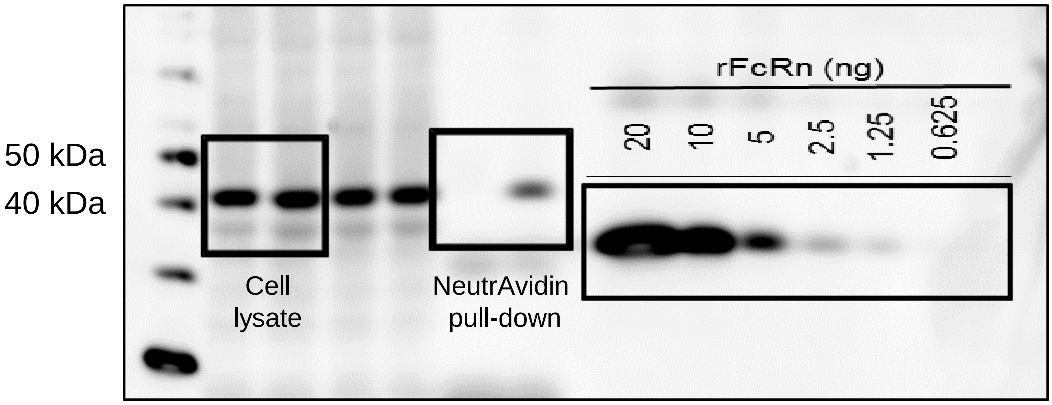

Supplementary Figure 1. Representative (uncropped) blot from which cropped images in Figure 2B and 2C are taken. Boxed regions correspond to cropped images in Figure 2. Note that the recombinant FcRn is the extracellular domain only and is therefore lower molecular weight than endogenous full length FcRn. This difference is accounted for in calculations.
